# The Minor Variant of the Single-Nucleotide Polymorphism rs3753381 Affects the Activity of a SLAMF1 Enhancer

**Published:** 2017

**Authors:** L.V. Putlyaeva, A.M. Schwartz, A.V. Klepikova, I.E. Vorontsov, I.V. Kulakovskiy, D.V. Kuprash

**Affiliations:** Engelhardt Institute of Molecular Biology, Russian Academy of Sciences, Vavilova Str. 32, Moscow, 119991, Russia; Institute for Information Transmission Problems (Kharkevich Institute) of the Russian Academy of Sciences, Bolshoy Karetny per. 19, bldg. 1, Moscow, 127051, Russia; Belozersky Institute of Physico-Chemical Biology, Lomonosov Moscow State University, Leninskie Gory 1, bldg. 40, Moscow, 119234, Russia; Vavilov Institute of General Genetics, Russian Academy of Sciences, Gubkina Str. 3, Moscow, 119991 , Russia; Faculty of Biology, Lomonosov Moscow State University, Leninskie Gory 1, bldg. 12, Moscow, 119234 , Russia.

**Keywords:** autoimmunity, B cells, noncoding polymorphism, transcriptional regulation

## Abstract

The *SLAMF1 *gene encodes CD150, a transmembrane glycoprotein expressed on the surface of T and B-lymphocytes, NK-cells, dendritic cells, and subpopulations of macrophages and basophils. We investigated the functional regulatory polymorphisms of the *SLAMF1 *locus associated with autoimmune processes, using bioinformatics and a mutational analysis of the regulatory elements overlapping with polymorphic positions. In the reporter gene assay in MP-1 and Raji B-cell lines, the enhancer activity of the regulatory region of the locus containing the rs3753381 polymorphism demonstrated a twofold increase upon the introduction of the rs3753381 minor variant (G → A) associated with myasthenia gravis. An analysis of the nucleotide context in the vicinity of rs3753381 revealed that the minor version of this polymorphism improves several binding sites for the transcription factors of FOX and NFAT, and RXR nuclear receptors. All mutations that disrupt any of these sites lead to a decrease in the enhancer activity both in MP1 and in Raji cells, and each of the two B-cell lines expresses a specific set of these factors. Thus, the minor variant of the rs3753381 polymorphism may contribute to the development of myasthenia gravis by modulating *SLAMF1 *expression, presumably in pathogenic B-lymphocytes.

## INTRODUCTION

The SLAMF1/CD150 receptor encoded by the SLAMF1 gene is a transmembrane glycoprotein of 70 kD expressed on the surface of various hematopoietic human and murine cells: B and T cells (at various stages of differentiation), dendritic cells, and subpopulations of macrophages and basophils [1, 2]. The activation of these cells, as well as the activation of monocytes and mast cells enhances the expression of *SLAMF1 *[1, 3-5]. In T-lymphocytes, SLAMF1 has a co-stimulatory effect on the antigen-specific CD28-independent proliferation and induces the synthesis of IFN-γ [6], whereas in B-lymphocytes, it induces and enhances the proliferation and synthesis of immunoglobulins [7]. SLAMF1 is also important for bi-directional T-B-cell stimulation. The SLAMF1 protein may serve as a receptor for the measles virus [2], participate in the process of recognition of Gram-negative bacteria, and the subsequent activation of macrophages to kill bacteria [8]. It was shown in murine models that disorders in the signaling pathway of this protein can lead to the development of autoimmune diseases and immunodeficiency conditions [9-11].

Today, the role of the four representatives of the SLAM/CD2 family in the development of autoimmune conditions is well-known. Changes in the nucleotide sequences of the *Ly9, Ly108*, *CD84, *and *CD244 *genes are associated with the initiation of autoimmune processes not only in murine models, but also on a limited cohort of patients. The presence of alternative alleles of the *Ly108 *gene in mice strongly affects central tolerance during the development of B- and T-cells, because the *Ly108 *gene is involved in the TCR-mediated stimulation of the key proapoptotic molecules BIM and FasL [12], the regulation of immunological tolerance, and cell cycle progression [13]. Furthermore, Ly108 and CD84, along with their adapter SAP (SLAM-associated protein), are involved in a bi-directional T-B-cell stimulation which is required for the formation of germinal centers [14]. It is known that single-nucleotide polymorphisms of the genes that encode selected members of the SLAM/CD2 family are associated with the risk of developing particular autoimmune diseases. It is known that the minor variant of rs509749 in the *CD229 *gene (Ly9, SLAMF3) alters the aminoacid sequence of the ITSM-motif of CD229, followed by a change in the receptor affinity to the SAP adapter, which in turn may lead to an increased risk of systemic lupus erythematosis [15, 16]. There is also evidence of an association between the heterozygous variant (GA) of the single-nucleotide polymorphism rs6427528 of the *CD84 *gene with a positive response to treatment with etanercept in patients with psoriasis and rheumatoid arthritis [17] and two polymorphisms of the *CD244 *(2B4) gene – rs3766379 and rs6682654 – with a progression of rheumatoid arthritis and systemic lupus erythematosus: this was established in a cohort of Japanese patients [18]. It is also known that the rs2049995 polymorphism in the *SAP *gene, encoding the basic adapter protein of SLAM family representatives, correlates with the development of systemic lupus erythematosus [19]. All this evidence is indicative of a relationship between changes in the nucleotide sequences of SLAM genes with the development of various autoimmune diseases.

**Table 1:** Polymorphisms of the SLAMF1 gene locus associated with autoimmune processes

SNP	rs11265455	rs3753381
SLAMF1 gene enhancer	D	E
Association with disease	Type 2 diabetes mellitus	Myasthenia gravis
Risk allele	G (minor)	A (minor)
Alternative allele	A (major)	G (major)
Frequency of risk allele	0.199	0.25
P-values	3.9 × 10^-5^	9.63 × 10^-6^
OR	1.32 (1.16–1.47)	1.04 (0.87–1.25)
TFBS, presumably destroyed by minor variant of SNP	BPTF	RXR, FOX

Note: OR – odds ratio for disease; risk allele/allele associated with the risk of disease; TFBS – transcription factor binding sites.

Two *SLAMF1 *polymorphisms associated with autoimmune processes are known *(Table 1)* [20, 21]. The results of genotyping described in these articles suggest that the minor variant of the rs11265455 polymorphism is associated with the risk of type 2 diabetes mellitus, while the minor variant of the rs3753381 polymorphism (G > A) is associated with an increased risk of myasthenia gravis.

According to data obtained in mice with induced obesity, type 2 diabetes, which has previously been known to be the only metabolic disease associated with impaired interaction between insulin and body tissue cells, also has an autoimmune nature [22, 23] and can develop concomitantly with other autoimmune diseases [24, 25]. The B cells involved in the glucose metabolism and activation of proinflammatory macrophages and T cells and the production of a unique profile of IgG autoantibodies in obese humans play an important role in the development of type 2 diabetes. It was shown in a mouse model of type 2 diabetes that anti-CD20-antibodies reduce T cell activation and improve glucose metabolism [22]: and The use of salicylates and IL-1 antagonists, which reduce the glucose level, passed clinical trials [26].

Acquired myasthenia gravis is a rare autoimmune disease which clinically manifests itself in fatigue and weakness of striated muscles [21], [27]. The trigger mechanism that activates the autoimmune response system in myasthenia gravis has not yet been established: autoantibodies against the nicotinic acetylcholine receptors located in the motor nerve termination area are produced, leading to impaired nerve impulse transmission to the muscle [28]. Injection of an immunoglobulin fraction from the serum of a patient with myasthenia gravis, comprising anti-AChR antibodies (found in 85% of patients) and anti-MuSK antibodies (in 15% of patients), induces myasthenia symptoms in animals [9, 29, 30]. In patients with myasthenia gravis, therapy aimed at reducing the amount of B cells using monoclonal antibodies against CD20 (rituximab) is effective [31]. Change in T-cell receptor (TCR) signaling, which in turn may affect the selection system in thymus, together with the activity of T helpers and regulatory T cells, is another known risk factor of development of autoimmune diseases [32]. The development of many autoimmune diseases, such as systemic lupus erythematosus, polymyositis, dermatomyositis, rheumatoid arthritis, Sjogren’s syndrome, multiple sclerosis, acquired epidermolysis bullosa, Crohn’s disease, ulcerative colitis, and autoimmune hepatitis, is associated with an impaired production of NKT-cells (Natural Killer T cell)[33]. There is evidence that a twofold increase in *SLAMF1 *expression in NOD.Nkrp1b.Tg (Slamf1) mice doubles thNKT-cells production in thymus by means of homotypic interactions (SLAM-SLAM) on the surface of immature NKT-cells and CD4+CD8+ thymocytes, which are required for the development of NKT-cells [9, 34]. A small increase in CD150-SLAMF1 expression also enhances the production of IL-4 and IL-17 in response to stimulation through the TCR [34]. There is evidence that *SLAMF1 *is involved in the regulation of IFN-γ production by CD4+ T cells, which can also be indirectly related to the pathogenesis and immune regulation of myasthenia gravis [3]. This suggests that an increase in the production of *SLAMF1 *induced by the allelic variant of a single-nucleotide polymorphism can be one of the links in the chain of autoimmune processes.

Recently, we described several regulatory regions that control the expression of the *SLAMF1 *gene, including the promoter (297-0 with respect to the translation start site) and three enhancer elements of approximately 2.5 kb, two of them located in the third intron and one at a distance of 3 kbp after the coding sequence [35]. The activity of these regulatory elements was studied in Raji and MP-1 cell lines (Burkitt lymphoma and acute lymphoblastic leukemia models, respectively). It was shown that the expression of *SLAMF1 *mRNA is controlled by the EBF1, SP1, STAT6, IRF4, NF-kB, ELF1, TCF3, and SPI1/PU.1 transcription factors, which bind to the promoter and enhancer regions.

This paper presents data on two additional enhancer elements of the *SLAMF1 *gene locus (hereinafter enhancers E and D*), *where two polymorphisms, rs3753381 and rs11265455, associated with autoimmune processes, are localized. We studied the effect of each of these polymorphisms on the expression of the *SLAMF1 *gene in B cells. The enhancer E comprising rs3753381 polymorphism is located in the third intron of the *SLAMF1 *gene*, *and enhancer D is located at a distance of 1.5 kbps before the coding region of the gene.

Our study of polymorphisms in the *SLAMF1 *gene locus showed that both the minor and major variants of rs11265455 have almost no effect on the activity of the *SLAMF1 *promoter*, *while the minor variant of the rs3753381 polymorphism (located in enhancer E) increases the activity of the *SLAMF1 *promoter more than twofold. We identified FOX, RXR, and NFAT as nuclear protein families whose binding depends on the allelic variant of rs3753381 and have pointed at the particular members of these families which are specifically expressed in the investigated cell lines (HNF4G, RXRB, and FOXO_2_ in the MP-1 and NFATC/3 and NR2C1 in Raji).

## EXPERIMENTAL

**Cell culture and transfection procedure**

MP-1 and Raji cells were cultured in a RPMI medium (PanEco) supplemented with 10% fetal calf serum, *L*-glutamine, antibiotics, essential amino acids, HEPES, and sodium pyruvate. Transfection was performed using the Neon Transfection System (Life Technologies, USA) at the rate of 2 × 10^6^ MP-1 cells and 7 × 10^6^ Raji cells per transfection. Luciferase activity was assayed after 24 hours using a Dual Luciferase Assay kit (Promega, USA).

**Plasmid Constructs**

Genetic engineering manipulations were carried out using standard techniques; enzymes produced by Fermentas/ThermoScientific (Lithuania) were used. In order to produce the constructs pGL3-rs3753381 (G) and pGL3-rs11265455 (A), sequences of the enhancers E and D, respectively, were amplified with primers containing the restriction sites SalI and BglII (only SalI in the case of enhancer D) and then cloned into a WT SLAMF1 vector [35] cleaved at the BamHI-NcoI sites, together with a fragment of the pGL3-basic vector cleaved at the SalI-NcoI sites. Constructs with alternative variants of the respective polymorphisms, rs3753381 (A) and rs11265455 (G), were produced on the basis of the pGL3-rs3753381 (G) and pGL3- rs11265455 (A) constructs. Mutations at the binding sites of the FOX and RXR proteins were introduced via site-directed mutagenesis of the core sequences of the sites using appropriate primers. Mutagenesis was performed using two-stage PCR, and the resulting constructs were purified using the NucleoBond Xtra Midi Kit (Macherey-Nagel, Germany) and verified by sequencing using the Sanger method. The nucleotide sequences of the primers are shown in *Table 2*.

**Table 2:** Oligonucleotide primers used in this study.

Primer	Nucleotide sequence 5’-3’	Application
E150–5Sal (for)	TTTTGTCGACCCTGTACCTTATTCT	Amplification of enhancer E and introduc- tion of SalI and BglII restriction sites
E150–5Bgl2 (rev)	TTTAGATCTATCCTTGCCTTAAGGC	
rs3753381-F	ATTTTTACAGAGTTCACAGCTTCCAGA	rs3753381(A) design
rs3753381-R	CTGTGAACTCTGTAAAAATGTTTACTTGGA	
S1enh7F	AGAAGAATTTGGGGGCAGAGAGGACT	Amplification of enhancer D and intro duction of SalI restriction site
S1enh7SalR (rev)	AAAAGTCGACCCGCCCTTTTTCATGAGTTAAAC	
for G RXRA	TACGGATTTATCAGCTTCCAGAAAA	mut RXR G design
rev G RXRA	AAGCTGATAAATCCGTAAAAATGT TTAC	
for A RXRA	TACAGATTTATCAGCTTCCAGAAAA	mut RXR A design
rev A RXRA	AGCTGATAAATCTGTAAAAATGT TTAC	
for G FOXO3	CATTACAACGGAGTTCACAGCTT	mut FOX G design
rev G FOXO3	CTCCGTTGTAATGTTTACTTGGATG	
for A FOXO3	CATTACAACAGAGTTCACAGCTT	mut FOX A design
rev A FOXO3	CTCTGTTGTAATGTTTACTTGGATG	

**Bioinformatics analysis of binding sites**

The genomic segments in the vicinity of the rs3753381 and rs11265455 polymorphisms of the *SLAMF1 *gene locus were analyzed using public ChIP-Seq data for the B-lymphoblastoid cell line GM12878 (which is etiologically similar to the MP-1 and Raji cell lines) available in the UCSC Genome Browser [36]. We considered the presence of the H3K27Ac histone mark, DNAse I accessibility [38] according to ENCODE DNase-Seq data for GM12878, and the presence of experimentally determined transcription factor binding sites as evidence of regulatory elements [39]. Prediction of transcription factor binding sites overlapping with polymorphic positions was carried out using the HOCOMOCO motif collection [40]. The effect of allelic variants on the the predicted binding affinity was assessed using the PERFECTOS-APE software [41] with the default settings.

**The analysis of differential gene expression**

The MP-1 and Raji samples analyzed in our study were obtained in [35]. The resulting sequencing reads are available in the NCBI Sequence Read Archive: project identification number is PRJNA313457.

## RESULTS AND DISCUSSION

**The minor variant of rs3753381 polymorphism increases the activity of the *SLAMF1* enhancer**

**Fig. 1:**
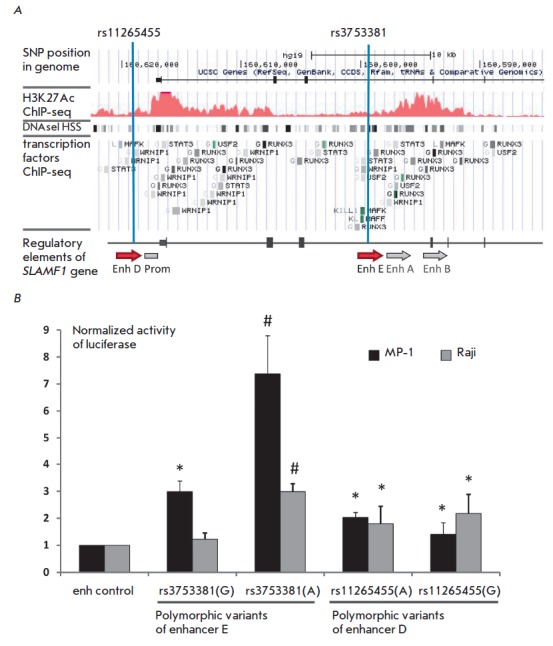
Analysis of the putative enhancers of the SLAMF1 gene locus. A. Schematic representation of the regulatory elements of the SLAMF1 locus. Grey arrows indicate enhancers A and B, as described previously [32]; thick black lines indicate SLAMF1 gene exons; thin lines indicate introns. Red histogram indicates the level of H3K27 acetylation, rectangles mark DNase I hypersensitivity clusters and transcription factor binding sites according to ENCODE ChIP-Seq data. Vertical blue lines schematically show the location of the SNPs rs3753381 and rs11265455. B. Effect of allelic variants of rs3753381 and rs11265455 on the activity of enhancers E and D. The bars correspond to the expression of the reporter gene in MP-1 and Raji cell lines, normalized to the activity of the construct containing the control fragment without enhancer activity [32]. All data are from three or more independent experiments. Data represent mean values ± SEM. “*” indicates a statistically significant difference between experimental and control constructs; “#” indicates a statistically significant difference between the construct containing the minor variant of the rs3753381 polymorphism and the construct containing the common variant (P < 0.05, Student’s t-test).

We have previously described the promoter and three enhancers of the *SLAMF1* gene, A, B, and C [35] (*Fig. 1A*, enhancers A and B are shown by gray arrows). In our study, we chose two additional alleged regulatory regions to analyze the possible impact of single nucleotide polymorphisms on the regulation of *SLAMF1* expression*:* rs3753381 polymorphism is located in the putative enhancer E (the third intron of *SLAMF1*), and rs11265455 polymorphism is located in the putative enhancer D, which is 1,500 bp upstream of the *SLAMF1* coding region *(Fig. 1A, *shown by red *arrows).* These regulatory elements were cloned in two stages (see Experimental) into a WT SLAMF1 vector [35]. All constructs stimulated *SLAMF1 *promoter activity*, *which confirms the function of D and E as potential transcription enhancers.

Next, single substitutions were introduced into the sequences of the enhancer elements D and E so as to replace the existing rs3753381 and rs11265455 alleles with alternative variants associated with the development of type 2 diabetes mellitus and myasthenia gravis, respectively (see *Table 1*).

Both putative enhancer elements increase *SLAMF1 *promoter activity (see *Fig. 1B*) compared to the previously described [35] control sequence, whose length is equal to that of the tested enhancer elements but is not enriched in transcription factor binding sites or H3K27 acetylation marks. It is noteworthy that the activity of enhancer E was significantly higher in the MP-1 cell line than in the Raji line, and enhancer D activity was low and was about the same in both cell lines. Since these cell lines are similar in terms of maturity and etiology, the difference in the activity of alleged enhancer elements, apparently, can be explained by differences in the transcription factors expression in MP-1 and Raji.

*Figure 1B* shows that the presence of a minor variant of rs3753381 polymorphism increases the activity of enhancer E in both cell lines, and the minor variant of rs11265455 polymorphism has no significant effect on the activity of enhancer D in any of the examined lines. Therefore, we proceeded with studying the rs3753381 polymorphism in more detail.

**Mutations at the RXR and FOX transcription factor binding sites reduce enhancer E activity in the case of minor variant of rs3753381 polymorphism**

**Fig. 2:**
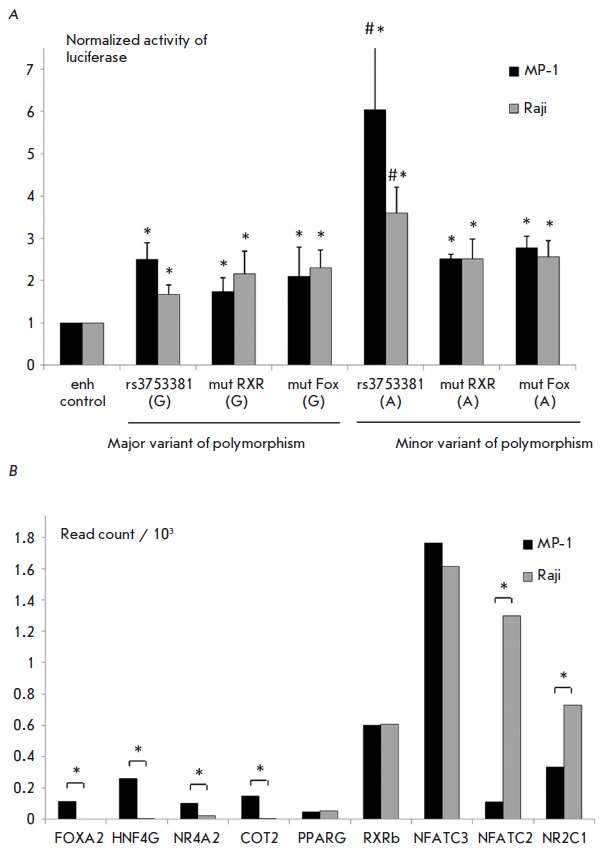
The influence of allelic variants of the rs3753381 polymorphism on the binding of transcription factors. A. The effect of mutations in the RXR and FOX binding sites on the activity of enhancer E. See Fig. 1B for legend. B. Expression of the transcription factors whose binding could be affected by the mut RXR and mut FOX mutations. The bars correspond to the normalized number of reads obtained from a RNA-seq analysis of MP-1 and Raji cell lines. “*” indicates a statistically significant difference between the samples (FDR < 0.05).

We performed a bioinformatics analysis of the transcription factor binding sites which could be affect ed by the studied polymorphisms in order to explain the significant increase in enhancer E activity when a minor variant (A) of rs3753381 polymorphism was introduced. We analyzed the sites that overlapped with rs3753381 and its allelic variant using the PERFECTOS-APE software. It was found that different members of the NFAT, RXR, and FOX families could bind to the rs3753381 polymorphism region and that their binding sites were stronger in the case of the minor variant (A). The predicted sites were mutated, and the effects of the mutations were tested in a system with a reporter gene *(Fig. 2A*).

**Fig. 3:**
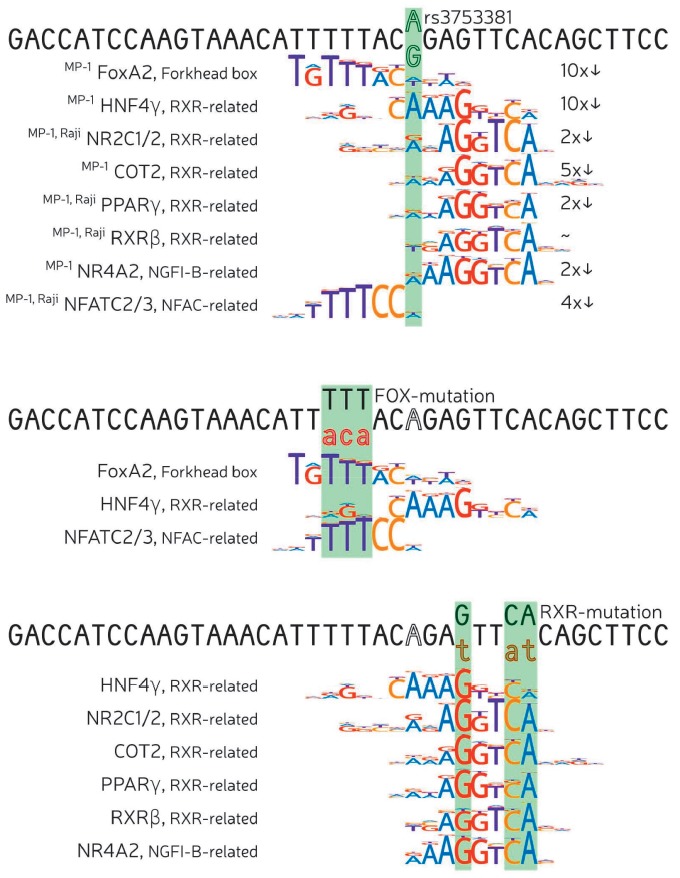
Alterations of binding site motifs by rs3753381 alleles and mutations of the RXR and FOX sites. The motif logos are shown under the corresponding segments of the enhancer E sequence. The predicted affinity loss for the major allele to minor allele substitution (G > A) is also shown.

Mutations in the RXR and FOX binding sites significantly reduce the activity of the enhancer with respect to the minor variant of rs3753381. The results were verified using a detailed bioinformatic analysis of the genomic sequence straddling the rs3753381 polymorphism. We selected the most reliable models of the binding sites from the HOCOMOCO database and conducted a joint analysis of all six sequence variants (rs3753381 (G), mut RXR (G), mut FOX (G), rs3753381 (A), mut RXR (A), mut FOX (A), using PERFECTOS-APE. We filtered only the predicted sites having a *P*-value of 0.001 or better for the wild-type sequence with the A allele. Then, we considered only those predictions where the affinity decreased or remained unchanged in all the alternative versions of the sequence (i.e., G allele or introduced mutations). Only the proteins expressed in the MP-1 and Raji cell lines were selected for further analysis *(Fig. 2B*, *Fig. 3*).

HNF4G, NR4A2, COT2, and PPARG are actively expressed in the MP-1 cell line, which may explain the significant decrease in enhancer E activity in the case of damaged binding sites of the aforementioned factors due to the mut RXR mutation *(Fig. 2A*, mut- RXR (A) and mut RXR (G) constructs). The Raji cell line is characterized by a high expression of RXRB and NR2C1, whose binding sites can be affected by the mut RXR mutation and, therefore, a disturbed binding of each of them can contribute to a reduction in enhancer E activity. As for the FOX factors, whose sites can be affected by the respective mutation, FOXA2 is highly expressed in the MP-1 cell line. NFAC (NFATC2 and NFATC3) proteins, which potentially bind to the same site, are expressed in the Raji cell line. Further, each mut FOX (A), mut FOX (G), mut RXR (A), and mut RXR (G) can affect the HNF4G binding site, and this causes a decrease in enhancer E activity in these constructs in MP-1 cells. When summarizing data on the mut RXR and mut FOX mutations, it can be assumed that the mut RXR mutation is associated with disrupted binding of HNF4G, RXRB, NR4A2, COT2, and PPARG in MP-1, and RXRB and NR2C1 in Raji, while the mut FOX mutation can disrupt the binding of HNF4G and FOXA2 in MP-1 and NFATC2, and NFATC3 in Raji. Thus, a change from the major variant of the rs3753381 polymorphism (G) to the minor rs3753381 (A) may change the binding of the RXR, FOX, and NFAC transcription factors, which are different in the case of MP-1 and Raji cell lines. The HOCOMOCO motif database covers only 600 of more than 1,500 human transcription factors [42]: We cannot exclude the possibility that other members of the aforementioned families likewise bind to the polymorphic region of the enhancer.

## CONCLUSION

Our study demonstrates the functional significance of the polymorphisms of the *SLAMF1 *locus associated with autoimmune processes, indicating a possible relationship between the rs3753381 and rs11265455 polymorphisms and the regulation of *SLAMF1 *gene expression*. *We explored the association between the minor variant of rs3753381 and the more than twofold increase in the activity of the *SLAMF1 *enhancer using the experimental model of human B-lymphoblastoid cell lines. The bioinformatics analysis of the sequences of the minor and major variants of the polymorphisms predicted that transcription factors of the NFAT, FOX, and RXR families likely contribute to the increase in enhancer E activity in the case of the minor variant of the rs3753381 polymorphism. It was shown that mutations in the predicted binding sites reduce the activity of the enhancer E carrying a minor variant of the rs3753381 polymorphism. It was also found that change in the allelic variant of the rs11265455 polymorphism has no significant impact on *SLAMF1 *gene expression*. *

## Abbreviations

SLAM: signaling lymphocytic activation molecule
CD: cluster of differentiation
IFNγ: interferon gamma
TCR: T-cell receptor
IL: interleukin
